# Toll-like receptors and their role in neuropathic pain and migraine

**DOI:** 10.1186/s13041-022-00960-5

**Published:** 2022-08-20

**Authors:** Xuejiao Liu, Wenping Yang, Chenlu Zhu, Songtang Sun, Shouyi Wu, Longde Wang, Yonggang Wang, Zhaoming Ge

**Affiliations:** 1 Department of Neurology, The Second Hospital of Lanzhou University, Lanzhou, China; 2 Gansu Provincial Neurology Clinical Medical Research Center, The Second Hospital of Lanzhou University, Lanzhou, China; 3 State Key Laboratory of Veterinary Etiological Biology and World Organisation for Animal Health/National Foot and Mouth Disease Reference Laboratory, Lanzhou Veterinary Research Institute, Chinese Academy of Agricultural Sciences, Lanzhou, 730046 Gansu China; 4 Expert Workstation of Academician Wang Longde, The Second Hospital of Lanzhou University, Lanzhou, China; 5 Headache Center, Department of Neurology, Beijing Tiantan Hospital, Capital Medical University, Beijing, China

**Keywords:** Migraine, TLRs, Microglia, Inflammatory response

## Abstract

Migraine is a complex neurological disease of unknown etiology involving both genetic and environmental factors. It has previously been reported that persistent pain may be mediated by the immune and inflammatory systems. Toll-like receptors (TLRs) play a significant role in immune and inflammatory responses and are expressed by microglia and astrocytes. One of the fundamental mechanisms of the innate immune system in coordinating inflammatory signal transduction is through TLRs, which protect the host organism by initiating inflammatory signaling cascades in response to tissue damage or stress. TLRs reside at the neuroimmune interface, and accumulating evidence has suggested that the inflammatory consequences of TLR activation on glia (mainly microglia and astrocytes), sensory neurons, and other cell types can influence nociceptive processing and lead to pain. Several studies have shown that TLRs may play a key role in neuropathic pain and migraine etiology by activating the microglia. The pathogenesis of migraine may involve a TLR-mediated crosstalk between neurons and immune cells. Innate responses in the central nervous system (CNS) occur during neuroinflammatory phenomena, including migraine. Antigens found in the environment play a crucial role in the inflammatory response, causing a broad range of diseases, including migraines. These can be recognized by several innate immune cells, including macrophages, microglia, and dendritic cells, and can be activated through TLR signaling. Given the prevalence of migraine and the insufficient efficacy and safety of current treatment options, a deeper understanding of TLRs is expected to provide novel therapies for managing chronic migraine. This review aimed to justify the view that TLRs may be involved in migraine.

## Introduction

### Migraine

Migraine is a neurological disorder that manifests as a paroxysmal headache lasting approximately 4–72 h. This type of headache may be unilateral and is characterized by pulsating or throbbing. It is generally associated with nausea and/or vomiting and sensitivity to light and sound. It can be relieved after rest and aggravated after activity. If treated inactively or improperly, headache severity may progress throughout an attack and even develop into chronic migraine [1].

Migraine is divided into episodic (< 15 monthly headache days, MHDs) and chronic (≥ 15 MHDs, with migraine attacks occurring at least 8 days per month), according to the frequency of headache days per month [2]. In the 2016 Global Burden of Disease Study, migraine was a leading cause of disability among patients under 50 years of age worldwide, second only to lower back pain [1, 3].

However, the exact etiology and pathogenesis of migraine are still under discussion, resulting in limited treatment options. Recent studies have shown that Toll-like receptors (TLRs) are significantly associated with migraine. They mediate inflammatory pain and cause central sensitization by generating inflammatory mediators (e.g., TNF-α, IL-1β, and NO) [4].

### Neuropathic pain

Neuropathic pain was redefined as pain caused by a lesion or disease of the somatosensory system [5, 6]. Its symptom severity and duration are often greater than those of other types of chronic pain [7], with 5% of patients debilitated despite the use of analgesics [8]. Therefore, in-depth study of the role of TLRs in neuropathic pain is conducive to better treatment. Several recent studies have demonstrated that TLRs are dramatically associated with neuropathic pain [4, 9–17]. Its pathogenesis may be that they induce the activation of microglia or astrocytes and the production of the proinflammatory cytokines in the spinal cord, resulting in the development and maintenance of inflammatory pain and neuropathic pain. In particular, primary sensory neurons express TLRs to sense exogenous PAMPs (pathogen-associated molecular patterns, PAMPs) and endogenous DAMPs  (damage-associated molecular patterns, DAMPs) released after tissue injury and/or cellular stress.

### History of TLRs

TLRs have been characterized by their essential contribution to innate immune signaling [18, 19]. They were first discovered in the form of genes in *Drosophila melanogaster* that control the dorsal-ventral axis during embryonic development [20]. Toll was further identified as a transmembrane interleukin-1 receptor homolog that initiates immune responses in *Drosophila* in vitro [21, 22]. A human homolog of *Drosophila* Toll (Toll-like) was cloned and characterized as a transmembrane protein that can activate nuclear factor-κB (NF-κB), mediating transcription of the proinflammatory cytokines IL-1, IL-6, and IL-8 in human monocytes [23]. The discovery of this receptor provided preliminary evidence that TLRs are regulators of mammalian immunity [18, 24].

### Structure and function of TLRs

**Fig. 1:**
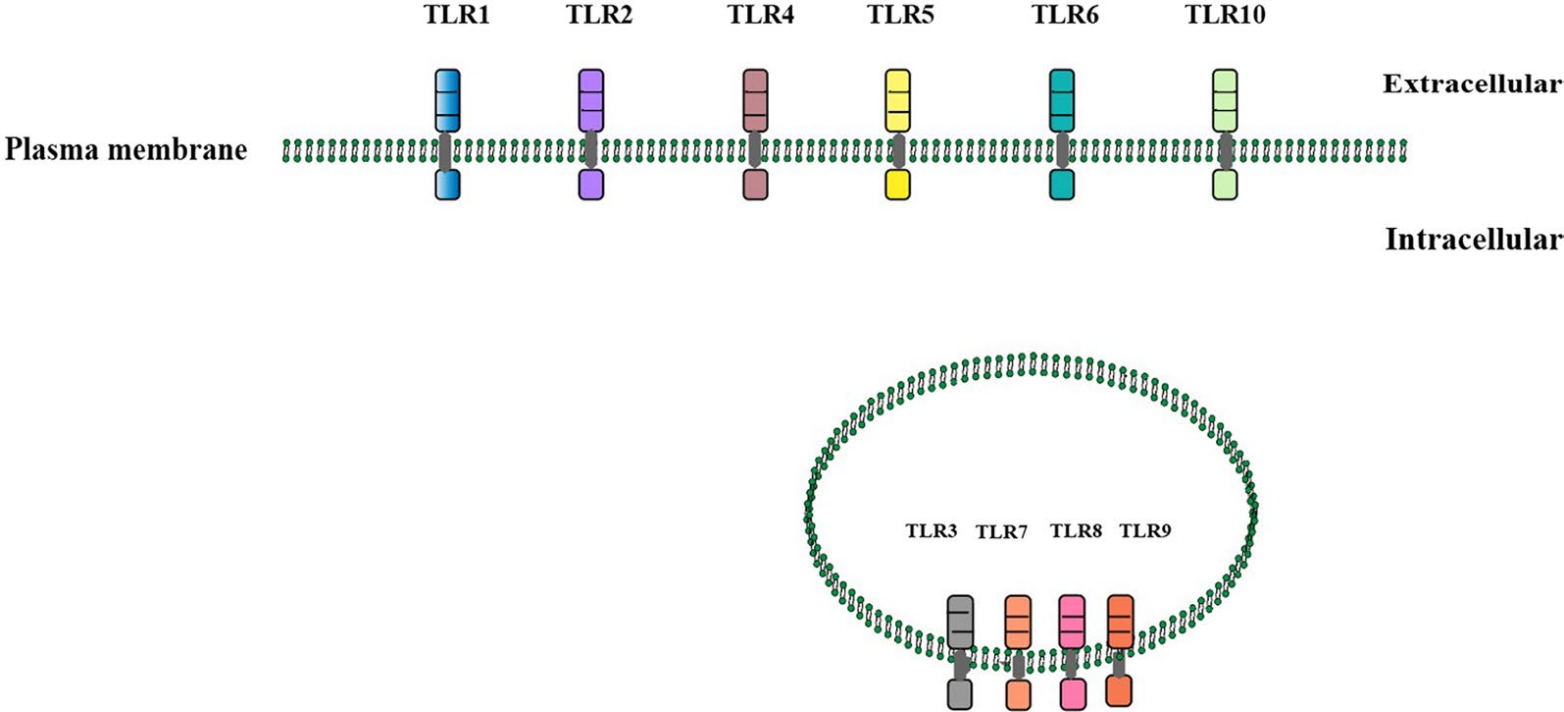
Location and structure of TLRs in cells. In human cells, TLR1, TLR2, TLR4, TLR5, TLR6, TLR10 reside at the plasma membrane, while the nucleic acid–sensing TLRs (TLR3, TLR7, TLR8, and TLR9) are localized within endosomal compartments

All TLRs consist of an amino-terminal domain that has multiple leucine-rich repeats and a carboxy-terminal TIR domain that interacts with TIR-containing adaptors. Thirteen TLRs have been identified in humans and rodents. Humans functionally express TLR1 to TLR10, whereas rodents express TLR1 to TLR9 and TLR11 to TLR13 [18]. TLR10 is the latest to be discovered [25]. TLR2 likely forms heterodimers with TLR1 and TLR6, whereas other TLRs form homodimers. Nucleic acid–sensing TLRs (TLR3, TLR7, TLR8, and TLR9) are located within the endosomal compartments, while other TLRs (TLR1, TLR2, TLR4, TLR5, TLR6, TLR10, TLR11, TLR12) reside at the plasma membrane [26–28] (Fig. 1).

The TLR gene was discovered to be one of the key genes during development. TLRs are specific type-I transmembrane receptors and pathogen pattern recognition receptors in the innate immune system. These receptors initiate immediate innate immunity by recognizing pathogens and can initiate adaptive immunity via activating signaling pathways. However, they are also expressed in many non-immune tissues, both throughout development and in adulthood. Several studies have indicated that TLRs not only exert immune functions, but also have a wide range of functions in regulating cell fate, cell number, and cell shape [29–33]. These receptors also play a key role in regulating the survival of nerve and glial cells and regulating synaptic plasticity in the central nervous system (CNS) [34].

### Signaling pathways of TLRs

The TLR ligands include exogenous pathogenic microorganisms and endogenous ligands released after tissue injury or damage. TLRs play an essential role in recognizing specific patterns of microbial components involved in the activation of innate immunity. Simultaneously, they can initiate a series of downstream reactions by binding to endogenous ligands during acquired immune activity. These noxious endogenous ligands are known as DAMPs (also known as alarmins).

**Fig. 2:**
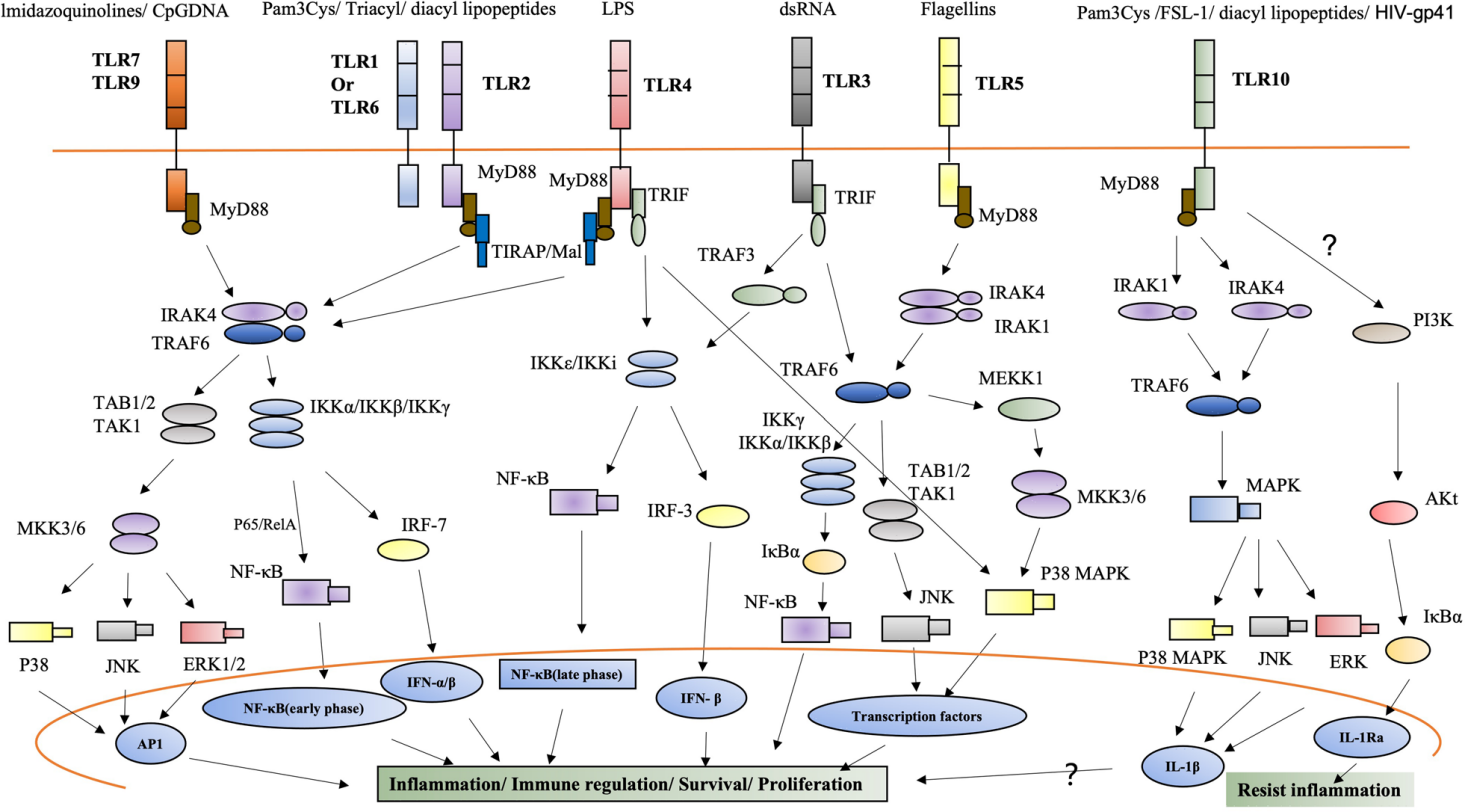
TLRs signaling. TIR domain-containing adaptors and TLR signaling. MyD88 is an essential TIR domain-containing adaptor for the induction of inflammatory cytokines via all the TLRs. Upon stimulation, MyD88 recruits IL-1 receptor-associated kinase (IRAK) to TLRs. IRAK is activated by phosphorylation and then dimerizes with TRAF6, leading to the activation of two distinct signaling pathways, finally activating MAPK and NF-kB to elicit proinflammatory cytokines. TIRAP/Mal is a second TIR domain-containing adaptor that specifically mediates the MyD88-dependent pathway via TLR2 and TLR4, While TRIF specifically participates in the MyD88-independent pathway mediated by TLR3 and TLR4, TLR2 leads to the complexity of signal pathway by forming tlr2-tlr1 and tlr2-tlr6 heterodimers and starts intracellular signal transduction. Both homodimers (TLR10/TLR10) and heterodimers (TLR10/TLR2) can recruit MyD88. TLR10 can reduce the production of IL-1β by directly inhibiting MyD88 or MAPK. Although several studies have suggested its inflammatory properties, TLR10 has also been shown to increase the production of IL-1Ra (an anti-inflammatory factor), but the underlying mechanism is still unclear, as indicated by question marks. Nucleic acids in endolysosomes activate TLR3, TLR7 or TLR9 and initiate different and overlapping signal cascades

TLRs signaling pathways arise from intracytoplasmic TIR domains, which are conserved among all TLRs [35]. Recent evidence has suggested that upon ligand binding, the cytoplasmic TIR domain of TLR recruits MyD88, TIRAP, TRAM, and TRIF (signal transduction connectors), which modulate TLR signaling pathways [26] (Fig. 1). In summary, different adaptors activate different kinases (IRAK4, IRAK1, IRAK2, TBK1, and IKKε) and ubiquitin ligases (TRAF6 and pellino 1), finally activating the NF-κB, type I interferon, p38 MAP kinase (MAPK), and JNK MAPK pathways [26, 36, 37] (Fig. 2).

MyD88 is essential for the induction of inflammatory cytokines triggered by all TLRs. TIRAP is specifically involved in the MyD88-dependent pathway via TLR2 and TLR4, whereas TRIF is involved in the MyD88-independent pathway that is mediated by TLR3 and TLR4. Thus, the diversity of TIR domain-containing adapters provides the specificity and complexity of TLR signaling [35]. The TLR5, TLR7, TLR8, and TLR9 signaling pathways are MyD88-dependent.

Research on TLR10 signaling is currently inconclusive. To date, TLR10 is the only TLR known to exhibit anti-inflammatory properties. Previously, TLR10 was thought to be an “orphan receptor,“ but many recent studies have identified ligands of TLR10 [25, 38]. Some studies have suggested that TLR10 activation can promote inflammation by activating NF-κB, while others have shown that it suppresses inflammation by inhibiting NF-κB. However, the downstream signaling pathway remains to be elucidated. The complexity of TLR10 signaling may be related to its ability to form TLR2/TLR10 heterodimers or TLR10/TLR10 homodimers.

### TLRs and migraine

TLRs are normally expressed in immune and glial cells of the CNS [39, 40]. In addition to pathogen recognition, TLRs also function to recognize the molecular patterns of ligands associated with cellular stress, tissue damage, or cell death [41–43].

Significant evidence has illustrated that innate immune signaling is the major mechanism responsible for persistent pain [18]. Previous studies have also suggested that TLRs (TLR2, TLR3, TLR4, TLR5, TLR7, TLR8, TLR9) are implicated in the pathogenesis of neuropathic pain models [4, 9–17]. However, the role of TLRs in migraines remains unclear. To date, several studies have shown that TLR2, TLR3, and TLR4 are associated with migraine [44–48].

**Fig. 3:**
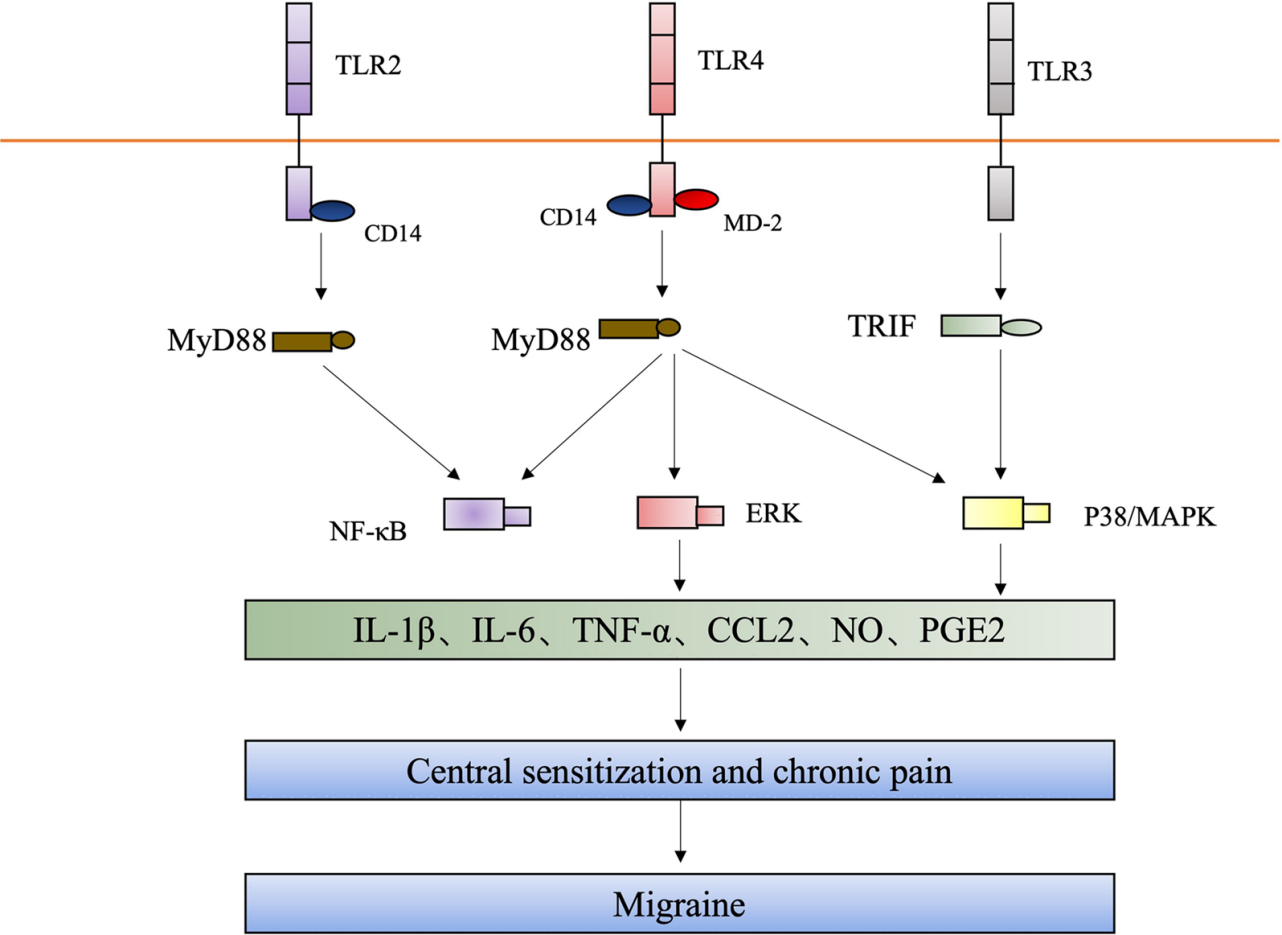
TLRs 2, 3, and 4 mediate migraine. Activation of TLRs (TLR2, TLR3, and TLR4) triggers upregulation of NF-κB and increases the transcription of genes encoding IL-1 family cytokines and TNF. Upon activation, a series of cytokines and inflammatory mediators are expressed that lead to central sensitization and possibly migraine

The possible mechanisms by which TLRs cause migraine are as follows: activation of TLRs leads to the upregulation of NF-κB, while increasing the transcription of genes encoding IL-1 family cytokines and TNF [49, 50]. After activation, Th1, Th2, and Th17 effector cells express a series of cytokines that act on innate immune cells to fight infections and may cause migraine [51–53] (Fig. 3).

## Discussion

### TLR2

#### TLR2 signaling

Residing at the plasma membrane, TLR2 is characterized by an exceptional diversity of compatible exogenous and endogenous ligands [18, 54]. This is mainly because it can dimerize with TLR1 or TLR6, which increases the complexity of ligand specificity. Structural studies have confirmed that TLR2 can distinguish various lipopeptides by forming TLR2/TLR1 and TLR2/TLR6 heterodimers [18, 55, 56]. Ligand-induced heterodimerization of the TLR2 extracellular domain brings the cytoplasmic C-terminal TIR domain into proximity and then initiates intracellular signaling via the MyD88 -dependent pathway. This then leads to upregulation of NF-κB and increased transcription of genes encoding the IL-1 family cytokines and TNF by binding to the cofactor CD14, which induces the production of inflammatory cytokines, resulting in pain [18, 51, 57].

In addition to reacting with exogenous ligands, TLR2 is triggered by its binding to endogenous ligands when the body is damaged and stressed. Although endogenous DAMPs for TLR2 have not been definitively captured, a few studies have shown that TLR2 can bind to high-mobility group box 1 (HMGB1), Ganglioside GT1b, Heat Shock Protein 60 (HSP60), biglycan (a type of CSPG), and hyaluronan [18, 58–62].

The TLR2 signaling pathways are complex, not only because it is easy to form a heterodimer, but also because there seems to be a large overlap between some endogenous ligands and their effects on TLR2 and TLR4, creating crosstalk between TLR2 and its downstream targets. The ability to directly attribute to functional results to TLR2 depend on the method used [18].

#### TLR2 and neuropathic pain

TLR2 is found in many organisms, where it induces the generation of inflammatory cytokines, activating NF-kB, with consequent pain [51, 63]. Although low levels were detected in astrocytes, oligodendrocytes, Schwann cells, fibroblasts, endothelial cells, and neurons, they are predominantly expressed on microglia and other macrophages in the peripheral and central nervous system [49, 50, 64–66].

Several studies have shown that TLR2 activates microglia and astrocytes and produces proinflammatory cytokines in the spinal cord following tissue and nerve injury, leading to the development and maintenance of inflammatory and neuropathic pain [4]. Several researchers have also found that Tlr2 knockout partially alleviated mechanical allodynia and thermal hyperalgesia caused by nerve ligation [12, 18, 67].

#### TLR2 and migraine

RNA sequencing of the brain revealed that Tlr2 gene expression is highly enriched in microglia compared to other cell types, and has been identified as a reliable marker of activated microglia in vivo, but its detailed role in microgliosis is still unknown [18, 68, 69]. Previous studies have suggested that TLR2 is involved in the pathogenesis of neuropathic pain and trigeminal neuralgia. However, the mechanism of TLR2 pathway during migraine attacks remains unclear [40].

Evidence suggests that TLR are significantly associated with migraine. Transcriptomics has demonstrated that the expression of proinflammatory genes (e.g., TLR2, CCL8) in the calvarial periosteum is significantly increased in patients with CM [44]. In a study of migraine with aura, multiple cortical spreading depression (CSD) episodes induced significant HMGB1 release, and the HMGB1-TLR2/4 axis activated microglia [45]. Several studies have shown further evidence that both mast cells and T cells are activated and the expression of chemokine and TLR2 are increased in migraines [51, 70, 71].

An increase in inflammatory cytokines leads to increased cell adhesion, production of chemical inflammatory compounds, and NF-κB dysfunction (Fig. 3). Therefore, reducing inflammatory symptoms in migraine may affect innate immune response pathways by modulating the inflammatory cytokines, TLRs and NF-κB [44].

It is not difficult to see that research on TLR2 and migraine is still in its infancy. Further work is needed to elucidate the upstream and downstream molecular mechanisms of migraine.

### TLR3

#### TLR3 signaling

TLR3 is an intracellular receptor localized within the endosomal compartments. In addition to DRGs, TLR3 is thought to be expressed to varying degrees in microglia, astrocytes, oligodendrocytes, Schwann cells, fibroblasts, and endothelial cells [18].

Intracellular TLR3 is intrinsically capable of detecting nucleic acids. It acts within the endosomal compartment and can distinguish between host and foreign nucleic acids. This role is exerted at specific stages of endosomal maturation and acidification.

TLR3 recognizes double-stranded RNA (dsRNA) and is MyD88-independent [72]. TLR3 is specific to dsRNA, and in addition to ligand dsRNA, TLR3 is also able to recognize some ssRNA viruses [27]. It is unique among all TLRs, and it signals through the TRIF pathway, resulting in the release of type I interferons via IRF3 and/or inflammatory cytokines via NF-κB [18, 36].

#### TLR3 and neuropathic pain

Research on the mechanism of its involvement in pain is increasing [27], and there is some initial evidence suggesting that TLR3 modulates pain through both shared and distinct molecular mechanisms. This is indirectly supported by the observation that DRGs express TLR3 in culture. TLR3 specific agonist (poly I: C) can increase TRPV1 expression and the functional activity of these sensory neurons, along with triggering an increase in the release of pro-nociceptive prostaglandin E2 [18, 73]. However, few studies have investigated the relationship between TLR3 and neuropathic pain.

A recent investigation identified elevated TLR3 mRNA and protein levels in the rat spinal cord after nerve injury along with increased activation of microglial autophagy. Intrathecal injection of the TLR3 agonist poly (I: C) significantly increased the activation of microglial autophagy and promoted neuropathic pain, which was dramatically reversed by TLR3 knockout [11].

Several studies have shown that TLR3 plays a substantial role in the activation of spinal microglia and development of tactile allodynia after nerve injury [74]. TLR3 deficient mice exhibit moderately reduced allodynia in response to nerve injury, suggesting that activation of TLR3 can be used to regulate neuropathic pain [12]. Tong Liu demonstrated a critical role of TLR3 in regulating sensory neuronal excitability, spinal cord synaptic transmission, and central sensitization. Central sensitization-driven pain hypersensitivity, but not acute pain, is impaired in Tlr3(-/-) mice [10]. However, the specific endogenous ligands of TLR3 and mechanisms by which they induce neuropathic pain remain unclear.

#### TLR3 and migraine

Although little research has been conducted on the relationship between TLR3 and migraine, there is direct and indirect evidence to suggest that TLR3 is associated with migraine. Research has shown that TLR3 mediates inflammatory pain and causes central sensitization. The specific signaling pathways are as follows: activation of TLR3 in spinal cord microglia results in the activation of the nuclear factor κB (NF-κB), extracellular signal-regulated kinase (ERK), and p38 signaling pathways, leading to the production of inflammatory mediators, central sensitization, and chronic pain [4] (Fig. 3).

However, there seem to be opposing conclusions regarding the association between TLR3 and migraines. Significant evidence suggests that TLR3 activation is neuroprotective and anti-inflammatory in CSD-induced neuroinflammation. Targeting TLR3 may be a novel strategy for developing new treatments for CSD-related neurological disorders [46].

This contradictory conclusion provides research space and innovation for future research. Apparently, research on the relationship between TLR3 and migraine is insufficient, and more research is needed in the future.

### TLR4

#### TLR4 signaling

TLR4 is one of the most widely characterized TLRs owing to its fundamental role in bacterial perception and the resulting inflammatory response. The canonical ligand for TLR4 is lipopolysaccharide (LPS). The recognition of LPS by TLR4 is multifaceted, and requires the coordination of multiple accessory proteins and coreceptors [18].

Among the TLRs, TLR4 is unique in its capacity to signal through both MyD88-dependent and TRIF-dependent pathways [18]. Conformational changes in TLR4 after binding to ligands recruit adaptor proteins (MyD88 and TRIF) to initiate intracellular signaling cascades. Its recruitment can lead to activation of NF-κB, MAPKs, activator protein-1 (AP-1), and IFN regulatory factor 5 (IRF5), culminating in the transcription of cytokines, chemokines, and other immune mediators [18, 75–78].

#### TLR4 and neuropathic pain

A growing number of studies have shown that TLR4 is a key receptor associated with persistent pain [18, 79–81]. The participation of the sciatic nerve in neuropathic pain was confirmed by drug interventions in a chronic contraction injury model [82]. The TLR4 antagonist LPS-RS reversed mechanical hypersensitivity in a mouse model of arthritis pain [83]. While antagonism of TLR4 may help prevent dysregulated pain, the involvement of TLR4 may help orchestrate some aspects of tissue repair in the context of nerve injury [18, 84, 85]. Therefore, targeting TLR4 in the treatment of neuropathic pain needs to be cautiously confirmed through further in-depth research.

#### TLR4 and migraine

The findings of Rafiei et al. suggested that TLR-4 polymorphism is a genetic risk factor for migraine [86]. Other evidence has indicated that TLR4 is associated with hyperalgesia in migraines. The TLR4 signaling pathway promotes hyperalgesia induced by acute inflammatory soup delivery by stimulating the production of proinflammatory cytokines and activating microglia [87]. IL-18-mediated microglia/astrocyte interactions in the medullary dorsal horn likely contribute to the development of hyperpathia or allodynia induced by migraines [88]. In periorbital hypersensitivity of migraine, the TLR4 antagonist (+)-naltrexone blocked the development of facial allodynia after supradural inflammatory soup [89].

In addition, the relationship between the gut microbiota and migraine is currently a hot research topic. Significant research has shown that migraine is associated with functional gastrointestinal disorders (FGIDs), such as functional nausea, cyclic vomiting syndrome, and irritable bowel syndrome (IBS). Modulation of the Kynurenine (l-kyn) pathway (KP) may provide common triggers for migraine and FGIDs involving of TLR, aryl hydrocarbon receptor (AhR), and MyD88 activation; Meanwhile, TLR4 signaling was observed to initiate and maintain migraine-like behavior through mouse MyD88, and KP metabolites detected downstream of TLR activation may be a marker of IBS. Therefore, TLR4 may play a role in the mechanism of migraine induced by FGIDS [47, 48] (Fig. 3).

Although the relationship between TLR4 and migraine is more well-studied than that between TLR2 and TLR3, the related upstream and downstream mechanisms still require significant research.

## Conclusions and perspectives

Decades of work have indicated that pain and inflammation are subtly entangled concepts. Here, we present evidence that TLRs are essential for migraine development. Research thus far has suggested that the TLR family members TLR2, TLR3, and TLR4 are associated with migraine, but the detailed underlying pathways and mechanisms remain unclear.

Since the effect of each TLR on pain varies widely due to its structure and cellular location, future studies should investigate the signaling properties of TLRs in migraine attacks at a deeper level, while seeking to translate preclinical insights into effective treatment. In the study of the relationship between TLR2, TLR3, TLR4, and migraine, more attention should be paid to the study of the detailed signaling pathways. We further dissected how each TLR affects nociception and how its expression in glial cells and neurons, or crosstalk between the two, differentially affects the processing of migraines. In addition to TLR2, TLR3, and TLR4, future research should also focus on the roles of TLR5, TLR7, TLR8, and TLR9 in the etiology of neuropathic pain in migraine. Despite these challenges, continuing to elucidate the role of each TLR in representing pain experience provides a very promising opportunity to improve pain in migraine sufferers.

## Data Availability

Not applicable.

## Abbreviations

CNS: Central nervous system
PAMP: Pathogen-associated molecular pattern
DAMP: Damage-associated molecular pattern
dsRNA: Double-stranded RNA
ERK: Extracellular signal-regulated kinase
HMGB1: High-mobility group box 1
IBS: Irritable bowel syndrome
LPS: Lipopolysaccharide
MAPK: MAP kinase
MHD: Monthly headache day
NF-κB: Nuclear factor-κB
TLR: Toll-like receptors
CSD: Cortical spreading depression
